# Marring Leishmaniasis: The Stigmatization and the Impact of Cutaneous Leishmaniasis in Pakistan and Afghanistan

**DOI:** 10.1371/journal.pntd.0000259

**Published:** 2008-10-29

**Authors:** Masoom Kassi, Mahwash Kassi, Abaseen Khan Afghan, Rabeea Rehman, Pashtoon Murtaza Kasi

**Affiliations:** Department of Pathology, Bolan Medical College, Quetta, Pakistan; Swiss Tropical Institute, Switzerland

Cutaneous leishmaniasis or “*Kal Dana*” (“the year-long sore”), as it is known locally, not only leaves a disfiguring scar on your face, but permanently changes your perception of your own self (Figure 1). “This is not me,” said the woman from Chaman, as she explained her encounter with the disease and the stigmatization associated with it.

**Figure 1:**
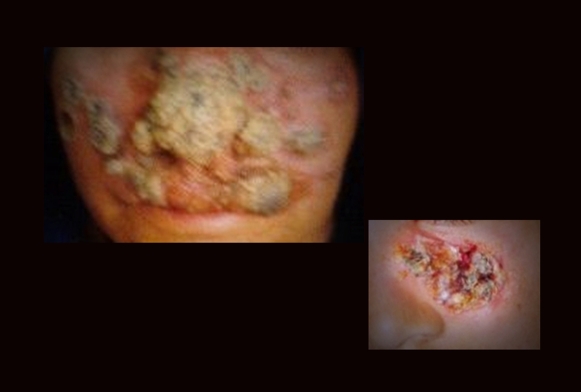
Marring leishmaniasis with lesions on exposed parts of the face.

This 28-year-old woman is a resident of Chaman, a town located near the Pakistan–Afghanistan border, in the southwestern province of Balochistan. For decades, Chaman has been a hub for refugees crossing the Durand line; it also is one of the many regions where poverty and lack of health resources show their true victimization of refugees. One such case is that of this woman.

She belonged to a poor family; her father, the sole breadwinner of the family, is a cattle rearer. She acquired her first lesion at the age of 12; from thereon she was socially excluded and not allowed to go to the local *madrassah* (an Islamic religious school). As she grew older, multiple other lesions appeared on her arms and face (the exposed parts of the body). She described this as a period of grief for her, when her parents started isolating her from the rest of the siblings to prevent further spread.

No treatment was acquired, due to the afflicting poverty and lack of resources of the region. But the scar had a deeper impact on this woman, as she is still unmarried in a community where early marriages are common. She describes her fate as, “I will probably get married to a limp or mentally disabled in a year or so; at least that is what happened to all the other girls who had these marks.”

## Which Parts of the World Are Affected by Cutaneous Leishmaniasis?

Globally, this disfiguring disease affects 1 to 1.5 million people. Approximately 90% of these infections occur in Afghanistan, Pakistan, Syria, Saudi Arabia, Algeria, Islamic Republic of Iran, Brazil, and Peru [1]. With respect to Pakistan and its neighboring war-torn country Afghanistan, the disease is endemic and the incidence is rising [2]–[5]. Outbreaks have been seen in Afghanistan and in refugee settlements along the border, including in northwest Pakistan during and after the Afghani crisis, with millions of refugees migrating into Pakistan [6]. In Pakistan especially, patchy epidemics have been seen in various locations from Balochistan to Multan to areas of Peshawar. In view of this distribution, leishmaniasis is embedded in poverty and underprivileged areas with limited access to health care.

With women and children being particularly affected by this marring disease, cutaneous leishmaniasis (CL) is now a major public health issue with considerable stigma associated with it [7]–[9].

**Figure 2:**
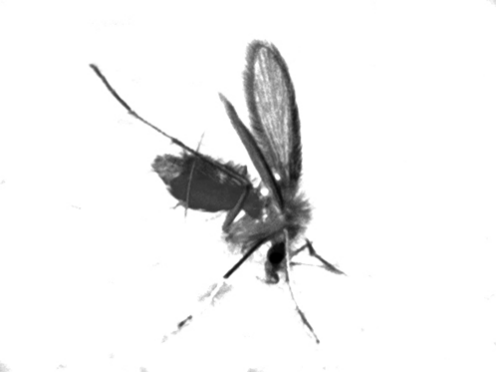
Sandfly—The vector of cutaneous leishmaniasis.

## How Is CL Spread?

Transmitted by the bite of the 2–3 mm-long sandfly (Figure 2), the disease is caused by more than 20 different species of the *Leishmania* parasite [1],[10]. The various species that produce CL include primarily *L. major*, *L. tropica*, and *L. aethiopica* in the “Old World”; and *L. infantum*, *L. braziliensis*, *L.* (V) *peruviana*, and *L.* (V) *guyanensis* in the “New World.” In Pakistan and Afghanistan, the most commonly reported species have been *L. tropica* [11],[12].

## How Is Social Stigma Created in Disease States?

As rightly pointed out by Peter J. Hotez in his recent editorial in *PLoS Neglected Tropical Diseases*, some of the so-called neglected tropical diseases not only cause “health and economic” effects but also result in “horrific social stigma” [13]. Although the morbidity associated with CL is not significant, and the disease is not lethal, the disfigurement and resulting social stigmatization may cause or precipitate psychological disorders, along with restricting social participation of the individuals affected by the disease. Thus CL, like other disfiguring diseases, not only affects the physical well-being of the individual but also significantly alters their psychological, social, and economic well-being [14].

A study conducted in five of the 14 districts of Kabul, the capital of Afghanistan, showed that many erroneous beliefs exist about CL, e.g., that “the disease can be transmitted by person-to-person physical contact” (of 360 respondents, the most common answers were “touching” [*n*  =  86] and “sharing meals and household goods” [*n*  =  26]). The study also indicated that “affected people are excluded from communal life” [15]. The level of exclusion varied from “minor domestic restrictions” such as not sharing plates to “severe physical and emotional isolation.”

People with active lesions and scars have been victimized as individuals and even in groups. Refugees from Afghanistan are identified by their “trademark” scars left behind by the disease.

As seen in the scenario presented, unaffected people tend to isolate affected people during the course of active lesions. Such lesions are a source of apprehension and even disgust. This indeed leaves a very strong impact on the individual as the scar lasts for years (“*Kal Dana*”—“the year-long sores”).

Due to the lack of awareness about the transmission of CL, people believe that the lesion is contagious. Patients are unnecessarily excluded from social groups. Although awareness is increasing, myths and wrong beliefs are still having an overwhelming impact.

## What Is the Impact of the Social Stigmatization on Individuals and Populations?

Stigmatization occurs in all age groups. During adolescence and childhood, parents tend to isolate their affected children from others in the family, including themselves. This surely is a dark period for a yet developing child who needs attention and care from the parent. Name-calling is sometimes seen. Unfortunately, in places where people are named at young ages according to some physical disability, these names sometimes persist for as long as they live.

Women are particularly victimized as they are considered unacceptable for marriage, sometimes by their own families [15]. Women are often separated from their children during the disease by their family and not allowed to breast-feed. These women usually suffer from depression and often anxiety.

The association of psychiatric disorders with CL has been reported in a study done in Sanliurfa, Turkey. Patients with an active lesion or healed scars showed increased rates of depression, anxiety, and body satisfaction impairment, while a larger decrement in the quality of life was reported in patients with active lesions [16].

Psychiatric disorders, such as suicidal intentions, decreased self-esteem, and those mentioned above have been known to be attributed to other dermatological problems such as acne [17]. But CL is particularly debilitating since the atrophic scar is carried for years and often does not decrease in size, let alone disappear with time. Furthermore, as noted above, the lesions occur on exposed parts of the body, mainly the hands and face [18],[19]. The facial lesions, affecting prominent features such as the nose or ears, are severely disfiguring and mould one's personality, completely changing one's perception of self. It is also noteworthy that social stigma is a significant factor contributing to psychiatric illness in skin diseases, along with disfigurement and discomfort.

## How Can Stigmatization Be Prevented?

We as authors believe that the attempt to decrease the burden and eliminate the social stigma of CL will need a multifaceted approach. Hence, learning from the experiences of similar diseases, such as leprosy, that cause stigmatization would be of immense value [20],[21].

The first approach would be to target the disease itself. CL would have to be eliminated or controlled. Measures to diagnose and treat the disease should be made both affordable and accessible. At the same time, measures to prevent CL should be given due emphasis. In the absence of an effective vaccine, “the first line of defense is to avoid sandfly bites” [22]. People need to be educated about the transmission of the disease and the different ways of controlling the vector. Many interventions, such as house spraying, insecticide-impregnated curtains, and insecticide-treated bed-nets, have been proven to be effective [23]–[25]. The need of the hour is to educate people about these measures, which are effective not only for CL, but also for other vector-borne diseases, such as malaria, that are endemic to the region. A participatory approach that mobilizes the community should be utilized when implementing any public health control measure in this region. Involvement of community leaders is an important feature, and they should be taken into confidence in implementing any interventional program. This would lead to greater compliance and sustainability of the intervention.

Secondly and perhaps even more importantly, there is a pressing need to educate people. As authors, we believe that this is very crucial. Unless people are educated (not merely literate), not only will the stigmatization of people with CL continue, but other problems, such as stigmatization and isolation of people with HIV or those who are addicted to drugs, will continue to prosper. As suggested by Reithinger and colleagues, a disease-specific health education strategy would have to be developed, thoroughly tested, and then implemented [15]. With little or no formal education available, especially to women in worn-torn Afghanistan, awareness regarding diseases endemic to the region needs to be increased. Programs aimed at promoting preventive measures such as use of insecticide-treated bed-nets or indoor house spraying with residual insecticides should be coupled with education and increasing awareness among the general public.

Posters and public health messages need to be everywhere in the country. Local general practitioners and other health care professionals should come forward and play an active role in educating the public. With media now available almost everywhere in the country and in every language, this media platform should be used more effectively to increase public awareness of diseases. School-based education should not be forgotten—it is equally if not more important to educate the youth to reduce the burden of stigma of CL and other diseases [26]. Thus, education and media campaigns would not only help in counteracting false beliefs regarding CL and other diseases, but would also raise awareness about new advances in the field.

However, it should be remembered that extensive literature on attempts to reduce stigma in other disease states has shown that knowledge/education alone is not enough. A multifaceted approach is what is needed, including increasing contact between people affected by CL and the community. The preventive and even the management programs should be at the level of the community, as such community-based programs would help “eliminate the social stigma in the patients' families” along with “educating the community” about CL [27].

Finally, socioeconomic development has to coincide with all these measures. Many neglected tropical diseases, especially some of the infectious diseases, were eliminated in the West long before effective treatment regimens were in place, mainly owing to socioeconomic development [28]. We must also empower people affected by CL, especially women, if their social stigmatization is to be reduced. The governments of both Pakistan and Afghanistan need to invest more into their education and health. The amount invested at the moment is petty when we compare Pakistan and Afghanistan to other neighboring countries. Pakistan faces many political and other social hurdles, and the problems are far worse in the neighboring war-torn Afghanistan. Although the obstacles are numerous and the task Herculean, effective strategies need to be applied and planning needs to be done, to improve the socioeconomic conditions of the people of these two countries.

With respect to stigma associated with CL and other diseases, it is very unfortunate that with effective diagnosis, treatment, and prevention strategies available, the disease still affects thousands across the country. The stigma further compounds the morbidity of the disease and leads to unfortunate psychological and social consequences like in the case of the woman from Chaman. We sincerely hope that this will improve in the very near future.

**Box 1. Learning Points**

- Globally, 1 to 1.5 million people are affected by marring cutaneous leishmaniasis, especially women and children.
- In Pakistan, CL is endemic and the incidence is rising, with the condition being far worse in neighboring war-torn Afghanistan.
- The marring and disfigurement that lead to social stigmatization are a cause for concern. Educating the public is key to removing the disease's stigma.
- Disease-specific health education programs need to be coupled with other preventive and management programs in endemic countries.
